# A Multi-Scale Directional Line Detector for Retinal Vessel Segmentation

**DOI:** 10.3390/s19224949

**Published:** 2019-11-13

**Authors:** Ahsan Khawaja, Tariq M. Khan, Mohammad A. U. Khan, Syed Junaid Nawaz

**Affiliations:** 1 Department of Electrical and Computer Engineering, COMSATS University Islamabad (CUI), Islamabad 45550, Pakistan; tariq_mehmood@comsats.edu.pk (T.M.K.); junaidnawaz@ieee.org (S.J.N.); 2 Biometric and Sensor Lab, Effat University, Jeddah 34689, Saudi Arabia

**Keywords:** directional filter bank, image segmentation, multi-scale line detector, vessel segmentation

## Abstract

The assessment of transformations in the retinal vascular structure has a strong potential in indicating a wide range of underlying ocular pathologies. Correctly identifying the retinal vessel map is a crucial step in disease identification, severity progression assessment, and appropriate treatment. Marking the vessels manually by a human expert is a tedious and time-consuming task, thereby reinforcing the need for automated algorithms capable of quick segmentation of retinal features and any possible anomalies. Techniques based on unsupervised learning methods utilize vessel morphology to classify vessel pixels. This study proposes a directional multi-scale line detector technique for the segmentation of retinal vessels with the prime focus on the tiny vessels that are most difficult to segment out. Constructing a directional line-detector, and using it on images having only the features oriented along the detector’s direction, significantly improves the detection accuracy of the algorithm. The finishing step involves a binarization operation, which is again directional in nature, helps in achieving further performance improvements in terms of key performance indicators. The proposed method is observed to obtain a sensitivity of 0.8043, 0.8011, and 0.7974 for the Digital Retinal Images for Vessel Extraction (DRIVE), STructured Analysis of the Retina (STARE), and Child Heart And health Study in England (CHASE_DB1) datasets, respectively. These results, along with other performance enhancements demonstrated by the conducted experimental evaluation, establish the validity and applicability of directional multi-scale line detectors as a competitive framework for retinal image segmentation.

## 1. Introduction

Retinal fundus images provide a key insight into the various entities within the human retina. Any abnormal changes in these features point towards the type and seriousness of numerous eye diseases such as Diabetic Retinopathy (DR) and Diabetic Maculopathy (DM), both of which are main contributors towards global blindness. These diseases stealthily manifest themselves, and are usually not diagnosed until they have progressed to more advanced stages where their treatment becomes both costly and ineffective. One of the dominating feature in fundus images is the vessel tree structure, referred to as vasculature. Therefore, accurate detection of vaculature is the primary step as its detection acts as a reliable biomarker towards classification of both the retinal features and any associated pathologies [1].

Recent advancements in medical imaging technology have guided the scientists in the image processing community to focus on analyzing these medical images so as to mimic the diagnostic process of a medical professional. The computerized analysis of bio-medical images has found numerous uses in different medicinal applications ranging from diagnosis, progression monitoring, and treatment. Every passing day sees advances in digital image processing with expansion in both efficiency and the precision of such methods [2]. The data in bio-medical images is generally unbalanced (disease appears in small percentage of dataset) [3,4], unstable (variations in surface response due to uneven illumination) [5], and characterized by a high degree of complexity [3]. In this kind of a scenario, automated extraction of bio-markers in medical images exhibits great potential towards exploiting big data analytics in improving medical practice, and synchronization of diagnosis and prognosis conventions.

A broken bone, for example, is visible through an X-Ray, so researchers have aimed at developing an adaptive threshold that can detect the abnormality intelligently without any human supervision. Detecting retinal features and any associated pathologies is a bit more subtle than X-Rays. Over time, numerous highly sophisticated adaptive filtering techniques have surfaced that can almost match the human expert in the detection of these anomalies. The knowledge gap is still vast in terms of both detection and validation of these complex-shaped pathologies that result in numerous eye diseases every year.

**Figure 1:**
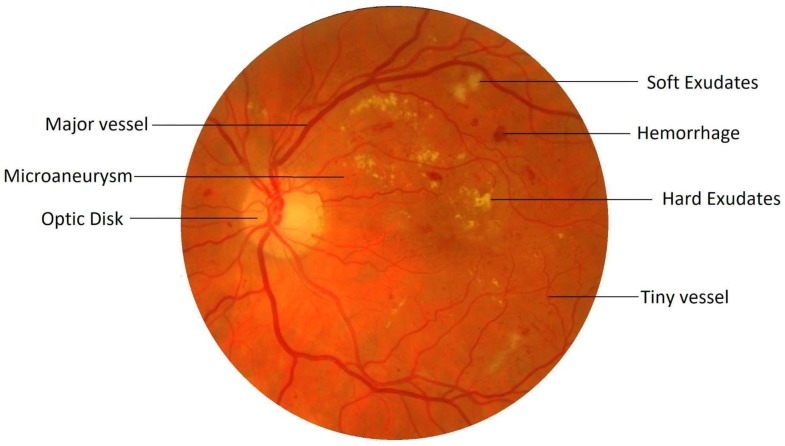
A retinal fundus image with features of interest.

Retinal image processing has attracted tremendous research focus over the years with varying degrees of success. Demarcation of the retinal vessel structure by a human expert is both time-consuming and tedious, thereby augmenting the need for automation of this crucial task to enable mass screenings. Pointing out troublesome features in the retina is a difficult task. For most of the other diseases, predefined lesion patterns help determine with conviction the presence or absence of a disease like cancer. However, DR is different as there can be multiple forms of anomalies associated with it, such as Microaneurysms, Exudates, and Neovascularization, etc., as shown in Figure 1. Each of these types is treated as a separate problem for detection and overall diagnosis. Then, the results from the integration of their outputs constitute the pathology detections in the image.

Automated vessel mapping of the retina is hindered by many factors, such as variations in the shapes, sizes, and direction of the dense vascular network present within the retina. Also, the enhancement of these blood vessels alongside the suppression of background information, plays an important role in increasing the accuracy of such a vessel mapping regimen. During the process of segmentation, all the existing morphological detectors tend to act poorly on vessels of varying sizes. The segmentation process becomes increasingly prone to error, particularly during the binarization phase, due to the changing orientation of vessels and poor contrast among them. Line detectors are one similar form of morphological detectors which have been employed for vessel detection in retinal images.

A basic or fixed-length line detector is based on establishing a correlation with the vessel geometry. A preset line segment is laid over a set of pixels, and a high matched response suggests the presence of a linear structure, or in this case, a vessel. Although line detectors are very efficient at detecting the presence of vessels, they fail to achieve an accurate segmentation due to the choice of length of the line detector. Detectors of smaller lengths or scales tend to produce shadows around vessel regions, and filters of larger length generate fake extensions at vessel endpoints. As there was a strict trade-off between these, this led to the evolution of multi-scale line detectors, which combines the responses of different scales while countering their adverse effects.

A multi-scale line detector identifies vascular structures in an image by iteratively rotating the detector at a pixel to capture the vessels at different angles at that point. With the rotation along some predefined angles, another critical parameter is the choice of detector’s variation in scale, which hunts for corresponding features of comparable lengths. However, this method of using multi-scale detectors had its own set of problems. This detector operates in all directions, and the efficacy of feature extraction in any specific direction is co-variant with the contrast of those features. While scanning multiple angles for the presence of vessels, the vessel features with high contrast dominate those with lower contrast. This results in the highlighting of certain vessel pixels and suppression of others to the extent that, tiny vessels, particularly at the endpoints of the vascular map, are usually the most affected. The tiny vessels are not the only victims to this problem, as the reconstruction of edges of the larger vessels are also affected during the combined binarization process.

To tackle these problems, we propose a three-pronged strategy with a new directional multi-scale line detector that, instead of scanning multiple angles within the whole image, focuses on a fixed direction while scanning a narrow angular range. Such a direction-specific filter will still be burdened by an image having multi-orientation features, thereby causing a loss in the sensitivity indicator. The second prong of this strategy lies in the use of directional images instead of the original image, having only those features or vessels which fall in line with the direction of the angular detector. This strategy is adopted with the expectation that, an image having only directional information will help boost the performance of the angle-specific detector while significantly reducing the false-positives.

Towards the end, a novel binarization technique based on the standard deviation of the directional line filter is used to eliminate the false extensions introduced by the larger-scale line filters. All the resultant features evolved from different directions, are recombined to yield the overall retinal vessel map. Therefore, directional segmentation and binarization of target features produces significantly improved results, as the detector becomes more immune to the omni-directional present in the input image. The proposed method is also notably faster as sensitivity, specificity, and accuracy of the vessel segmentation are enhanced without the use of any denoiser or advanced thresholding techniques such as region growing or overlaying.

The remaining portion of this paper is organized as follows: Section 2 summarizes the related work followed by Section 3, describing each step of the proposed technique in detail. Section 4 contains the experimental results elaborating the tested retinal image databases, algorithm’s performance judging criteria, and comparison with state-of-the-art techniques. Section 5 concludes the paper by discussing the key findings of this study, followed by recommendations for potential future research work.

## 2. Literature Review

Pointing out the features and troublesome lesions in the retina is a challenging task. Many research groups around the globe have been studying techniques to help determine these retinal features, and to maximize progress and collaboration, it has been turned into international competitions such as the STructured Analysis of the Retina (STARE) [6] and Digital Retinal Images for Vessel Extraction (DRIVE) [7] databases, etc. Many algorithms have surfaced that although not being applied in clinical practice yet, have provided unique insights into the solution of the problem at hand. A usual concern with any automated system is the accuracy and precision of the data it learns from. So, retinal images with all their differences in vessel size, contrast, presence of pathologies, and noisy vessel boundary problems make vascular segmentation an increasingly daunting task.

The vessel detection approaches using image processing, are categorized as either supervised or unsupervised learning techniques. Supervised methods identify the vessel pixels based on machine learning algorithms that use expert-marked ground truth samples to train a classifier. Unsupervised methods are the more popular choice, as they require no such pre-labeled training data and patterns of blood vessels hard-coded into the algorithm’s structure to obtain accurate segmentation.

### 2.1. Supervised Learning Methods

These techniques require a set of training data and relevant feature extraction followed by classification for vessel segmentation. First of all, constraints such as a viable training set (manually labeled into foreground and background), and the number of training data sets available, dictate the efficiency of the classification. Secondly, a feature index is created that helps differentiate between the vessel and non-vessel pixels which are later utilized by the classifier for the overall segmentation of vessels. The requirement of training data, large feature index and considerable classifier training time are the main drawbacks of supervised methods. On the other hand, ample training data coupled with a powerful classifier can lead to a significantly accurate segmentation of blood vessels.

Niemeijer et al. [8] used a multi-scale Gaussian feature vector followed by a K-Nearest-Neighbors (KNN) classifier for supervised vessel segmentation. Staal et al. [7] built a feature set evolved from 27 different attributes using the abstraction of image ridges, and fed them to a KNN classifier for calculating the probability of a pixel being a vessel entity. Ricci and Perfetti [9] used two orthogonal line operators and SVM on the green channel of retinal images to improve the accuracy and Area Under the Curve (AUC) parameters of their method. They reduced the false-positives and false-negatives by assignment of different weights to the errors of both categories, respectively. Marin et al. [10] utilized a grey-level and moment invariants-based feature set to train a multi-layer Neural Network (NN) to differentiate between the vessel and non-vessel pixels. You et al. [11] constructed a Support Vector Machine (SVM) and a feature vector by using radial projections from large and tiny vessels separately to recognize the retinal vasculature.

Fraz et al. [12] used an ensemble classification-based technique relying on orientation and morphological analysis of vessels. A multi-scale Gabor filter helped extract these features and a decision tree classifier was used in this approach, but it failed to report any objective quality measures. Franklin et al. [13] used all three-color planes of a fundus image to extract vessel features, eventually classifying through a multi-layer perceptron NN. Li et al. [14] proposed a cross modality learning approach without involving any preprocessing steps to induce robustness towards the training set. Their method was tested on DRIVE, STARE and CHASE_DB1 datasets with excellent results for both normal and pathological retinal fundus images.

Orlando et al. [15] used fully connected conditional random field method that worked with a structured SVM to achieve a high specificity score of 0.9870. They randomized the training process by taking random sample of pixels from different sets of images from the STARE dataset for efficient elongated structure segmentation through a machine learning approach. Dasgupta and Singh [16] also employed a FCCN to segment the vessels as a multi-label inference task. They used CLAHE during the preprocessing stage followed by 7 CNN layers on only the DRIVE dataset, to achieve remarkable sensitivity and accuracy scores of 0.9691 and 0.9533, respectively.

Alom et al. [17] used a Recurrent Residual Convolutional Neural Network (RRCNN) to take advantage of both residual and recurrent CNN for training deep architectures and better feature representation, respectively. They tested their technique on a wide variety of bio medical images including retinal fundus images with the same degree of efficiency. Oliveira et al. [18] used multi-scale Fully Convolutional Neural Networks (FCNN) that employ varying angles and branching patterns of vessels as the basis for data augmentation and prognosis. The Stationary Wavelet Transform explores the vessel rotation information to give the multi-scale tortuosity features that help the learning phase during training to refine the vascular map.

Li et al. [19] constructed a new DR dataset known as DDR, in which they compiled 13,673 retinal images from a wide variety of fundus imaging devices and hospitals across China. Many recent deep learning models were tested on this dataset to evaluate their efficacy in vessel and small lesion recognition. Li et al. [20] employed a connection sensitive loss model along with a attention gate NN to improve the accuracy of vessel detection. Segmenting thin vessels and boundaries was made more efficient by concatenating attention weights to features as this method reported an accuracy of 0.9563 and 0.9673 for the DRIVE and STARE datasets, respectively.

Jiang et al. [21] used extensive pre-processing and denoising for image cleanup, followed by a combined dilated convolution technique for data augmentation. A Deep Convolutional Neural Network (DCNN) was constructed with the use of skip layer connection during decoding, which helped their method achieve an impressive accuracy score of 0.9709. Guo et al. [22] proposed the use of multi-level features through a multi-scale Deeply Supervised Network (DSN) to achieve high AUC scores on the DRIVE, STARE and CHASE_DB1 datasets. The method used deep supervision by information exchange among higher and lower level layers for better fusion result, leading to effective retinal vessel segmentation and noise suppression.

Considering the disadvantages of supervised learning methods, a set of pre-labeled data, significant training computation times and complex feature vectors reduce the overall system efficiency in a real-world scenario. Also, the features of interest must be manually fed to the classifier as compared to the unsupervised learning methods, where the features are acquired by the algorithm itself. This causes a problem when the classifier is presented with a feature it is not already trained for. Therefore, the system may put that feature in the wrong class, or in this case, label a vessel pixel as non-vessel and vice-versa.

### 2.2. Unsupervised Learning Methods

These methods try to identify features of interest based on the similarity criteria and intrinsic attributes, without the need for manual intervention. These methods owe their popularity in literature to the independence from training data and faster execution times and therefore, learning is limited or altogether absent. Vessel detection in unsupervised methods is done at individual pixel-level, and the complicated nature of retinal vascular map combined with the presence of noise and pathologies implies that reliable segmentation remains a challenging problem. Unsupervised methods are further categorized into five groups: morphological approaches, matched filtering algorithms, vessel tracking, multi-scale processes, and model-based techniques.

Miri and Mahloojifar [23] presented the use of Fast Discrete Curvelet Transform (FDCT) alongside multidimensional morphological processing for retinal vasculature extraction. Vessel boundary was tediously revised utilizing a layered morphological system, where the wrongly detected boundaries were deleted during this revision process, allowing for efficient reconstruction of small and tiny vessels. Xu et al. [24] used elongation-based morphological shape detectors of retinal vessels by employing a twin attribute system that used geometric morphology alongside connected-tree branching methods, resulting in prominent vessel contrast enhancement.

Moghimirad et al. [25] proposed the detection of midpoints of a vessel through the weighted medialness 2-D functions, resulting in high accuracy figures for vessel radius evaluation in a broad range of noisy and pathological images. Nguyen et al. [26] suggested multiscale line identification, relying upon varying the length of a fundamental line detector to extract a threshold vessel image. A localized thresholding filter was stated by Ravichandran et al. [27] for detection of vessels built on the entropy of that region. Contrast Limited Adaptive Histogram Equalization (CLAHE) followed by Wiener filter was used to denoise the image followed by a 2-D Gabor filter for additional vasculature refinement.

Martínez-Pérez et al. [28] employed Region Growing (RG) technique to populate a scale-space representation of varying contrast via derivatives. The first and second derivative predictors helped cater for the contrast variation in the input image, resulting in superior segmentation results. Sofka et al. [29] segmented retinal vessels by developing a Likelihood Ratio Vesselness (LRV) value to assess the worth of matched filter responses. Kar S and Maity S [30] exhibited superior accuracy figures by using Curvelet function based on Fuzzy C-Means (FCM) architecture to detect blood vessels and the Optic Disc (OD). Amin and Yan [31] presented a robust vessel identification method in the form of sorted log-Gabor wavelets. The high paced wavelets exhibited noticeable immunity against the contrast and phase variations through a calibrated threshold.

Palomera-Pérez et al. [32] suggested a collateral setup which used a single plane (horizontal) and blended plane (horizontal plus vertical) feature markers for the evaluation of information within the retinal images. This parallel setup allowed for faster and effective feature extraction, and RG based vascular segmentation with a high degree of accuracy. Cinsdikici and Aydin [33] designed the combination of matched filtering with ANT colony algorithms to extract the entire vessel map. Some preprocessing followed by length sifting is utilized for vessel and capillary recognition, which became unproductive in cases with pathology.

Zhang et al. [34] used wavelet transform to convert standard images into 3D rotating orientation scores, which were further refined by multi-scale second-order Gaussian derivatives. This method was tested on six different retinal datasets and showed competitive results, especially with difficult cases like vessel crossings. Neto et al. [35] aimed to tackle the central vessel reflex by Gaussian smoothing and top-hat transformation. A curvature analysis followed by adaptive local thresholding resulted in excellent all-round performance parameters on both DRIVE and STARE datasets. Karn et al. [36] constructed a hybrid active contour model based on a Gradient Vector Flow (GVF) framework to focus on tiny blood vessels. A novel preprocessing technique coupled with phase-based binarization helped this method report high accuracy scores and effective vessel segmentation.

Aguirre et al. [37] utilized the removal of the OD and low-pass radius filter in preprocessing before employing a 30-element Gabor filter for vessel segmentation. Morphology-based decision rules and fractional derivatives helped this method achieve competitive performance parameters on the DRIVE database. Sundaram et al. [38] used a hybrid vessel segmentation algorithm based on morphological operators coupled with multi-scale vascular refinement. They used bottom-hat transform alongside fusion of resultant multi-scale images to tackle discontinuities at the boundaries of vessels. This method reported significant improvement in detection results, particularly on the High-Resolution Fundus (HRF) database but under-performed on the DRIVE and CHASE_DB1 databases.

## 3. Proposed Method

**Figure 2:**
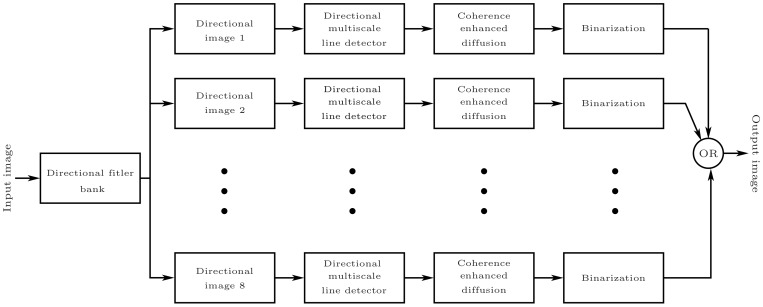
Block diagram of proposed method.

The main goal of this study is to enhance vessel detection by employing an improved version of multi-scale line detector, with emphasis on tiny vessels whose detection causes the most false positives during segmentation. Figure 2 depicts the overall flowchart of the proposed technique. The major building blocks of the proposed methodology are outlined as follows:

1. Creation of direction-specific image array using bandpass Directional Filter Bank (DFB) on the green channel of a RGB retinal image.
2. Employing multi-scale line detection at the orientated image array.
3. Using the Coherence-Enhancing Diffusion (CED) technique to enhance the sharpness of the segmented directional vessels.
4. Individual binarization of these directional images followed by linear recombination to efficiently threshold the vascular structures.

### 3.1. Bandpass Directional Filtering for Direction-Specific Vessel Isolation

Orientation-selective linear filters can mirror the localized flow of the direction of lines in a sub-region of interest, such as ridge patterns and retinal vascular networks [39]. Directional Filter Bank (DFB) is a similar kind of structure, which divides an image into corresponding set of orientation-field images. Each subsequent orientation-selective image relates to a discrete set of line segments with a narrow radial range. The frequency response of such an oriented bandpass filter can be further decomposed into its radial (ρ) and angular (ϕ) components by the following equation:

(1) $$H\left(\rho ,\phi \right)=H_{\mathrm{radial}}\left(\rho \right)H_{\mathrm{angular}}\left(\phi \right).$$

**Figure 3:**
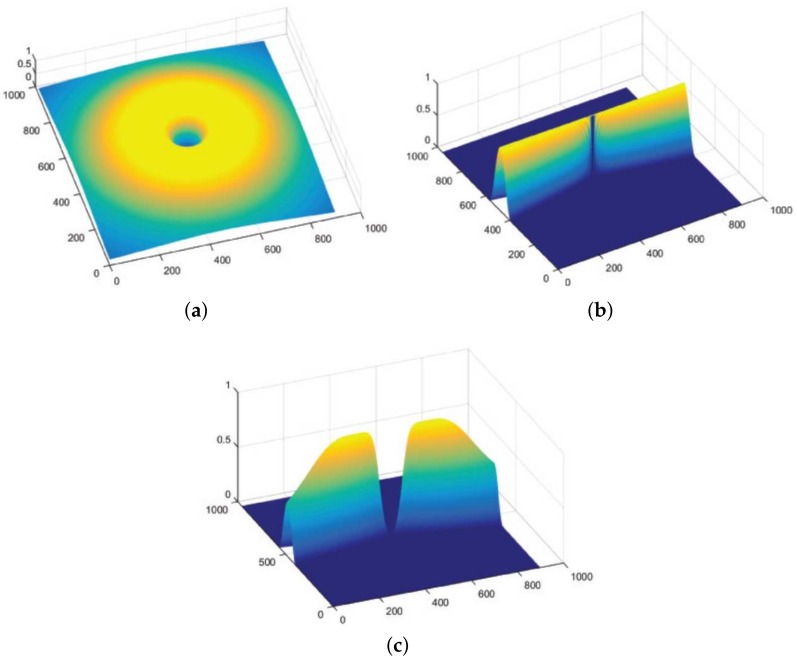
Setting up of the directional bandpass filter: (**a**) Exhibits $H_{\mathrm{radial}}\left(\rho \right)$; (**b**) Shows $H_{\mathrm{angular}}\left(\phi \right)$; (**c**) Depicts the complete bandpass directional filter H(ρ,ϕ).

Butterworth filter is selected to evolve $H_{\mathrm{radial}}\left(\rho \right)$ as:

(2) $$H_{\mathrm{radial}}\left(\rho \right)=\sqrt{\frac{{\left(\rho_{\mathrm{BW}}\right)}^{2n}}{{\left(\rho_{BW}\right)}^{2n}+{\left(\rho^{2}-\rho_{o}^{2}\right)}^{2n}}}\;,$$

where $\rho_{\mathrm{BW}}$ denotes the bandwidth or the angular width. $\rho_{o}$ depicts the center frequency, and n=2 gave suitable results for the experiment. $H_{\mathrm{angular}}\left(\phi \right)$ was set up by a directional kernel suggested by Knutsson et al. [40] as follows:

(3) $$H_{\mathrm{angular}}\left(\phi \right)=\left\{\begin{array}{cc} \cos^{2}\left(\frac{\pi \;(\phi -\phi_{c})}{2\;\phi_{\mathrm{BW}}}\right) & ;\;|\phi -\phi_{c}|<\phi_{\mathrm{BW}},\\ 0 & ;\;\mathrm{otherwise}, \end{array}\right.$$

where $\phi_{\mathrm{BW}}$ is the filter’s desired bandwidth. The span of desirable orientations is set in such a way that $|H_{\mathrm{angular}}\left(\phi \right)|\geq 0.5$, following which $\phi_{\mathrm{BW}}=\pi /k$, where *k* represents the decomposed distinct directional images. $\phi_{c}$ sets the filter’s orientation such that $\phi_{c}=i\pi /k,i=1,\cdots ,k$. The scope of these directional filters is from 0 to π as they sum to unity. An example of a directional filter H(ρ,ϕ) is shown in Figure 3. To deal with the retinal images, radial center frequency is set to a pixel width of 30, with a bandwidth of 60 pixels for vessel segmentation. In other words, a span of π/16 is the preset angular width iteration with center frequencies placed in the middle of these bandwidths at π/32,3π/32, and so on.

**Figure 4:**
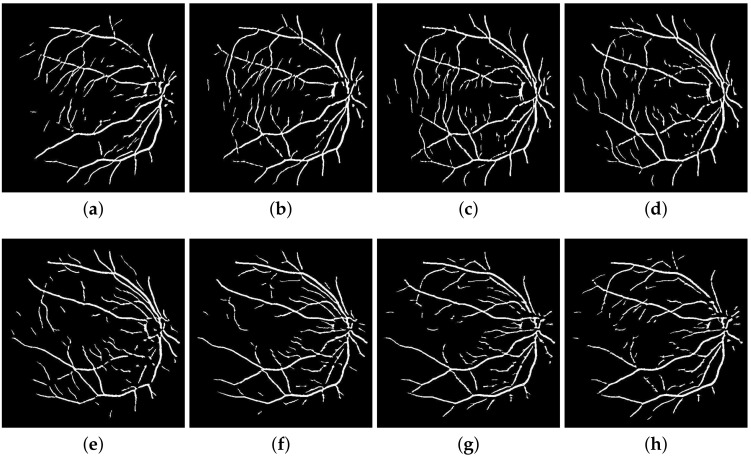
Directional image array built using the Bandpass Directional Filter Bank: (**a**) through (**h**) depict vessel responses corresponding to 8 iterations of 22.5 degrees from 0 to 180 degrees.

A retinal image f(x,y) is fragmented into its constituent directional filter bank involving numerous images $f_{i}\left(x,y\right)$, where i=1,2,⋯,n. Here *i* denotes the iterative increase in angle of the directional images spanning from 0 to 180 degrees. For our experiment, these orientations are given by $\theta_{1},\theta_{2},\cdots ,\theta_{8}$ which cover the complete angular width at an interval of 22.5 degrees, thus constituting a total of 8 images. Following this regimen leads to the constituent directional images with a high vessel response in only the specified orientation and a dull response elsewhere. DFB technique thus helps decompose an input retinal image into a diverse set of directional images, thereby highlighting all vessels corresponding to their specific angles only. The directional filter bank similar to Figure 3 was employed to help develop the direction-specific vessel images shown in Figure 4.

### 3.2. Multi-Scale Line Detector

A rudimentary line detector works by matching the average grey-level response of a possible vascular segment with 12 scanning lines oriented at 15 degrees to each other. This 180-degrees scan investigates a sub-image of a particular window size (usually 15 pixels). The line with the highest intensity response is labeled as the ‘winning line’, which depicts the presence and orientation of a vessel whereas lower responses confirm their absence. Therefore, any arbitrary pixel is classified as either a vessel or non-vessel pixel depending upon its variance from the winning line.

Another critical issue where line detectors excelled over other techniques, is the problem of central light reflex. It occurs when the edges of vessels exhibit a heightened intensity as compared to their mid-lines. This reflex causes a lot of false positives as the pixels near the center of vessels get misclassified as background. Line detectors, on the other hand, remain largely unaffected due to the winning line methodology. However, there are some major drawbacks with the basic line detector, the most important of which is its tendency to combine close vessels. Also, vessel crossover points induce false vessel augmentations, and edges of major vessels cause misclassification of background pixels as foreground.

To mitigate these negative effects, Nguyen et al. [26] suggested varying the scales of the scanning lines to distinguish vessel and background pixels, a technique he named as multi-scale line detectors. This was done by adjusting the length of the scan line *L* from 1 to the window size *W*, with the expectation that this would help alleviate the problems discussed above. Two intensity responses labeled as $I_{\max}^{L}$ (maximum line response), and $I_{\mathrm{avg}}^{W}$ (average window response) were used to calculate the multi-scale line detector as:

(4) $$M_{W}^{L}=I_{\max}^{L}+I_{\mathrm{avg}}^{W}.$$

This detector works brilliantly in all three problems faced by the basic line detector but reducing the detector’s line length introduces noise artifacts. This addition of noise comes from a narrower range of line detectors, leading to a weaker contrast among vessel and non-vessel pixels. To enhance the contrast, these raw response values are recombined in linear order to standardize these values to zero mean and unit standard deviation distribution as follows:

(5) $$M^{\prime }=\frac{M-M_{\mathrm{mean}}}{M_{\mathrm{std}}},$$

where M’ and *M* are the standard and raw responses, respectively. The statistical values of $M_{\mathrm{mean}}$ and $M_{\mathrm{std}}$ (mean and standard deviation of the raw values, respectively) help keep the distribution of intensity values in check. Therefore, these intensity values are extended over a broader range to significantly improve the contrast between the vessels and background, thereby reducing false positives.

Previously, the multi-scale line detectors were used to scan all the angles, and were not focused on any single direction. The method still worked remarkably well at detecting retinal vessels, but output response varied in contrast, making the detection process harder with a single threshold. This was due to the reason that, for an area having both types, the high contrast vessels dominated the detector’s focus and the weak contrast vessels got suppressed. Also, during the combined binarization process, tiny vessels, especially of 1-pixel width got wiped out. Not only the tiny vessels, but this difference in contrast also affected the major vessels as their borders were misclassified and thus narrowed down. Major vessels are often overlooked, but they are important as well when considering the overall efficiency of the segmentation process. For a major vessel having a width of 10 pixels, if the detector classifies 8 vessels correctly, that is still a loss of 2 pixels. Now looking at the percentage of major vessel pixels as compared to the tiny vessels in a retinal image, this loss of 2 pixels becomes a huge cumulative loss for the detector’s sensitivity.

**Figure 5:**
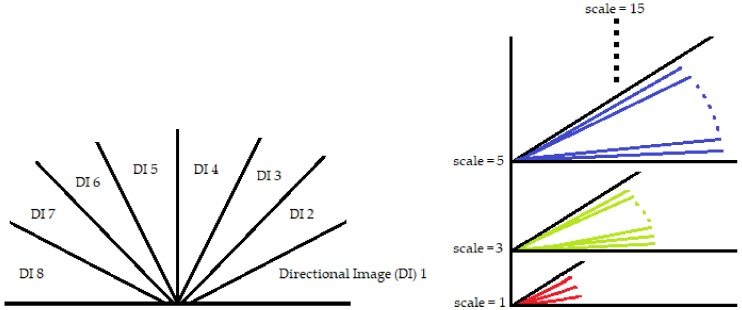
Left image shows 8 directional images of 22.5 degrees each, where every such image gets scanned by 11 line detectors of similar orientation while being scaled from 1 to 15 as shown on the right.

**Figure 6:**
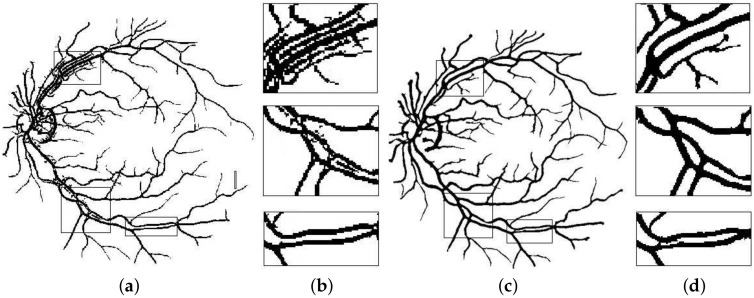
Segmentation results of: (**a**) Basic line detector; (**b**) Poor response at major vessels and vessel crossings; (**c**) Proposed method; (**d**) Improved response at critical points. This shadow removal happens largely due to alignment of line filter orientation with that of directional image it is operating on.

**Figure 7:**
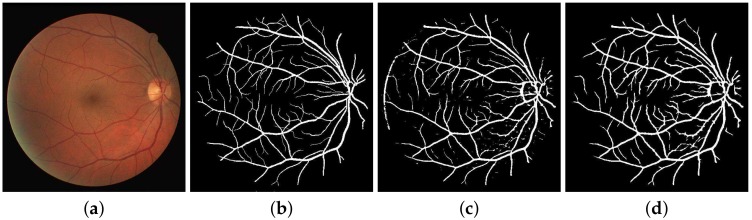
Response of directional multi-scale line detector: (**a**) Shows the original image; (**b**) Shows the ground truth; (**c**) Depicts the response of simple multi-scale line detector; (**d**) Shows the response of directional multi-scale line detector.

Here lies the novelty claim in this method such that, multi-scale detectors are made to operate in a narrower directional range instead of the whole 180 degrees. Not only this, the images these detectors work on are also directional in nature as acquired from the DFB technique as shown in Figure 5. This is done with the anticipation that, when both the image and filter’s directions are aligned, the filter’s response would be far superior, and features will be picked up more efficiently as depicted in Figure 6. To top it all off, the binarization is also done within these distinct directions to achieve improved vascular segmentation as shown in Figure 7.

The multi-scale line responses of every individual pixel are linearly recombined as:

(6) $$M_{\mathrm{combined}}^{d}=\frac{1}{n_{L}+1}\left(\sum_{L}M_{W}^{L}+I_{\mathrm{igc}}\right).$$

Combined responses $M_{\mathrm{combined}}^{d}$ of each of the 8 directional images are calculated separately where *d* depicts the direction. These directional responses go through the binarization process before being accumulated into the final segmented image. The window size is taken as 15 in which each scale is averaged using the same weight. The parameter $n_{L}$ represents the scale depth which is iterated from 1 till 15 with a step size of 2, thus resulting in a total of 8 scales. $M_{W}^{L}$ represents individual scale responses calculated from Equation (5), and $I_{\mathrm{igc}}$ is the inverted green channel response of each pixel in the RGB image. The parameter $I_{\mathrm{igc}}$ is included because the green channel information offers better contrast of the vessels as compared to other background entities such as OD and lesions [26]. In an original green channel, the vessels are dark on a light background, whereas this paper makes use of inverted green channel where vessels are light on a dark background for better visibility. This linear recombination scheme results in the reduction of background noise in the proximity of vessels.

### 3.3. Coherence-Enhancing Diffusion (CED)

Regularization of variations in elongated structures is done by applying anisotropic diffusion through a diffusion matrix, also known as Coherence-Enhancing Diffusion (CED). This filter is nonlinear but isotropic in nature and is dictated by the diffusivity parameter which allows or denies the diffusion at every point of an input image. This diffusion is done based on the feature’s orientation at that point in such a manner so as to avoid smoothing across the edges and thus, preserve sharpness in the image. In other words, the diffusion or smoothing is to be guided parallel to the edges rather than over them. The efficiency of this process is monitored through the development of the Orientation Field (OF) on a point-to-point basis and thus, an accurate OF is crucial to the whole CED regimen. The diffusion matrix calculates the diffusivity parameters by observing the local image structure alongside the alignment towards clean directions at each diffusion step. Each valid change is noted in a secondary matrix known as a second-moment matrix (or structure tensor), which is an established method for enhancing flow-like structures. Assuming a retinal image L(x,y), the anisotropic scale-space for L(x,y) is defined as:

(7) $$\partial_{t}L=\nabla \left(D\nabla L\right),$$

where *D* is the diffusion matrix of order 2×2. This diffusion matrix is adjusted to the area under consideration by the symmetric structure tensor μ, defined as:

(8) $$S=\left(\begin{array}{c} s_{11}\;\;\;\;s_{12}\\ s_{12}\;\;\;\;s_{22} \end{array}\right)=\left(\begin{array}{c} L_{x,\sigma }^{2}\;\;\;\;L_{x,\sigma }^{}L_{y,\sigma }^{}\\ L_{x,\sigma }^{}L_{y,\sigma }^{}\;\;\;\;L_{y,\sigma }^{2} \end{array}\right),$$

where $L_{x}^{2}$, $L_{x}L_{y}$ and $L_{y}^{2}$ represent the Gaussian derivative functions in both axes. The tuning parameter α is defined as:

(9) $$\alpha =\sqrt{{\left(s_{11}-s_{22}\right)}^{2}+4s_{12}^{2}}.$$

Using this parameter, the two dominant eigenvalues become:

(10) $$\begin{array}{c} \mu_{1}=\frac{1}{2}\left(s_{11}+s_{12}+\alpha \right),\;\\ \mu_{2}=\frac{1}{2}\left(s_{11}+s_{12}-\alpha \right). \end{array}$$

The expression ${\left(\mu_{1}-\mu_{2}\right)}^{2}$ comprehensively depicts the local variation of the gray values around a locality as follows:

- $\mu_{1}=\mu_{2}=0=0$; constant areas.
- $\mu_{1}\gg \mu_{2}$ or $\mu_{2}\gg \mu_{1}$; straight edges.
- $\mu_{1}=\mu_{2}\gg 0$; corners.
- $\mu_{1}=\mu_{2}\approx 0$; flat regions.

The last pieces of the puzzle are the eigenvectors which are later integrated with the eigenvalues to reconstruct the diffusion matrix *D*. One is the normalized eigenvector ${\left(\cos\theta ,\;\;\sin\theta \right)}^{T}$, and the other is the orthogonal eigenvector ${\left(-\sin\theta ,\;\;\cos\theta \right)}^{T}$. The eigenvalues of the diffusion matrix are taken as:

(11) $$\begin{array}{c} \lambda_{1}=c_{1},\;\\ \lambda_{2}=\left\{\begin{array}{cc} c_{1} & ;\;\mu_{1}=\mu_{2},\;\\ c_{1}+\left(1-c_{1}\right)\exp\left(\frac{c_{2}}{{\left(\mu_{1}-\mu_{2}\right)}^{2}}\right) & ;\;\mathrm{otherwise}, \end{array}\right. \end{array}$$

where $0<c_{1}<<1$, and $c_{2}>0$. The diffusion matrix *D* can be recreated with its eigenvalues and eigenvectors as:

(12) $$d_{11}=\lambda_{1}\cos^{2}\theta +\lambda_{2}\sin^{2}\theta ,$$

(13) $$d_{12}=\left(\lambda_{1}-\lambda_{2}\right)\sin\theta \cos\theta ,$$

(14) $$d_{22}=\lambda_{1}\sin^{2}\theta +\lambda_{2}\cos^{2}\theta .$$

Overall, the diffusion process advances in the following four pivotal steps:

1. Evaluate the second-moment matrix according to Equation (8).
2. Eigen-directions are computed using Equation (10), and diffusion matrix is constructed using Equations (12), (13), and (14).
3. Calculating change in intensity of each pixel based on its local neighborhood according to partial differential equation as $\nabla \left(D\nabla L\right)$.
4. Update image using the diffusion equation:
  (15) $$L^{t+△t}=L^{t}+△t\times \nabla \left(D\nabla L\right).$$

### 3.4. Binarization

It is a thresholding technique that converts a gray-scale image to a binary image and therefore, is a crucial step in the effective segmentation and classification of foreground features. A threshold value dictates this binarization phase, where a value greater than a carefully calibrated threshold allocates that pixel position to foreground and other values are classified as background or vice versa. As discussed during the line-detector phase, different lengths of detectors produce different artifacts (shadows and extensions) within the image. Method in [26] opted for multi-scale detectors as the detector had to move in all directions from 0 to 180 degrees. When a small length filter is aligned across a vessel, it produces shadows which cause false positives. This is not the case with our directional filter as the direction of the line filter is limited. However, the problem of extensions, which is caused by the larger scale filters still remains and is dealt with using traditional Niblack image thresholding [41].

Extensions can be avoided by using a better binarization scheme that involves the line filter’s standard deviation. A 2-D fissure likelihood measure [42], used to guide binarization process is defined as:

- If $P_{LR}$<$P_{mean}$−*k*×$P_{std}$: Classify pixel as background (this step removes the extensions).
- Else: Leave pixel unchanged.

**Figure 8:**
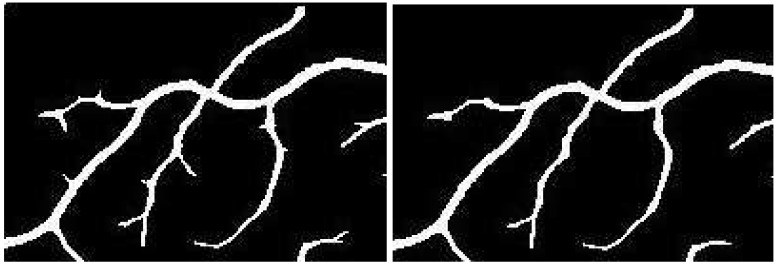
Removal of extensions: (**a**) Image shows extensions caused by large line filters; (**b**) Extensions removed by the Niblack adaptive threshold.

Here, $P_{LR}$ represents the line response of any arbitrary pixel while $P_{mean}$ and $P_{std}$ depict the line filter’s mean and standard deviation at that point, respectively. The bias parameter *k* is used to stabilize the impact of standard deviation from vessels of different sizes and Niblack proposed its value to be kept at −0.2. However, due to the sensitivity of the binarization process to this parameter, its values were tested from the range of −0.3 to 0.3, and the value of −1.5 was found to be suitable for this experiment. Figure 8 depicts the effectiveness of this step towards mitigating the effects of extensions created by the line filter.

The extension removal process is followed by the Linde–Buzo–Gray (LBG) algorithm for scalar values [43]. However, the initial cluster information is fed with Otsu method [44] to avoid trapping in local minimum. The steps for LBG algorithm for our application are as follows:

1. Input pixel intensity values $S=\left\{x_{i}\in R|i=1,2,\ldots ,n\right\}$.
2. Initialize a codebook *C*=$\left\{C_{0}\in R\right\}$ using Otsu method.
3. The initial codebook is split to create two values as $C_{1}=C_{0}-△$ and $C_{2}=C_{0}+△$, where △ is a small positive number.
4. Classify the image pixels into two clusters according to $x_{i}\in S_{q}$ if $∥x_{i}-C_{q}∥\leq ∥x_{i}-C_{j}∥$ for j≢q.
5. Update cluster centers $C_{j}$, j=1,2 by:
  (16) $$C_{j}=\frac{1}{\left|S_{j}\right|}\sum_{x_{i}\in S_{j}}x_{i}.$$
6. Set k←k+1 and compute the distortion as:
  (17) $$D_{k}=\sum_{j=1}^{2}\sum_{x_{i}\in S_{j}}∥x_{i}-C_{j}∥.$$
7. If $\frac{\left(D_{k-1}-D_{k}\right)}{D_{k}}>\epsilon$ (a small positive number), repeat step 4 to step 6.
8. The final threshold value is:
  (18) $$T=\frac{C_{1}+C_{2}}{2}.$$

## 4. Experimental Results

This section presents the conducted experimental results. A comprehensive analysis on the achieved results is also presented in this section.

### 4.1. Materials

Three standard publicly available databases: DRIVE [7], STARE [6], and CHASE_DB1 [45] were used for experiments in this work.

- **Digital Retinal Images for Vessel Extraction (DRIVE)**: part of a screening program in 400 diabetic retinopathy patients, aged 25–90 years in the Netherlands. It has 20 test and training images with a resolution of 565×584 pixels
- **Structured Analysis of the Retina (STARE)**: 20 images having a resolution of 700×605 pixels where half are normal, and half have pathologies such as hemorrhages, exudates and microaneurysms.
- **Child Heart And health Study in England (CHASE_DB1)**: 28 images acquired from both eyes of 14 multiethnic children in England. It also boasts a high resolution of 999×960 pixels.

The DRIVE and CHASE_DB1 datasets have distinct image sets to test and train the detector so the efficiency of the segmentation techniques can be judged. However, the manually segmented vessel tree for STARE dataset is only available for 20 images, and these were used as test images to evaluate the proposed methodology. Also, the Field Of View (FOV) images are given with the DRIVE dataset, but are not available for STARE and CHASE_DB1 datasets. The FOV for these two datasets was created using the image segmenter tool in MATLAB.

### 4.2. Evaluation Criterion

To gauge the efficiency of the proposed method, a resultant segmented image is compared with its corresponding ground truth. The ability to differentiate between vessel and non-vessel or background pixels, determines the effectiveness of a method at mapping the retinal vessel structure. Considering only the pixels inside the image’s FOV, classifications in both ground truth and segmented images were compared. This comparison results in two correct and two incorrect classifications. Segmentation results labeled as “positive” point towards foreground or vessel pixels, and “negative” suggests background or non-vessel pixels. Similarly, the phrase “positive” depicts a correct segmentation of a pixel while “negative” suggests a misclassification. Following this convention, True Positive (TP) is a pixel correctly classified as a vessel, and a False Positive (FP) is background pixel mistakenly classified as vessel. A True Negative (TN) is a pixel correctly identified as background, and a False Negative (FN) is a vessel pixel classified as background. These four possible results contribute towards the calculation of the following benchmark-specific test sets based on the interpretation of classifier decisions as:

(19) $$\mathrm{Sensitivity}\left(\mathrm{Sn}\right)=\frac{TP}{TP+FN},$$

(20) $$\mathrm{Specificity}\left(\mathrm{Sp}\right)=\frac{TN}{TN+FP},$$

(21) $$\mathrm{Accuracy}\left(\mathrm{Acc}\right)=\frac{TP+TN}{TP+FN+TN+FP}.$$

Sn depicts the ability of a method to segment vessels effectively while Sp represents background classification strength. Acc is a global ratio operator defined as pixels correctly classified as either vessel or background pixels divided by all pixels within the image. Area Under the Curve (AUC) is another commonly used parameter that is used to score binary classification methods. AUC integrates the area under the Receiver Operating Characteristic (ROC) curve to give a value of 1 for a perfect classifier and 0.5 for a totally random classifier. This is not applicable to this technique, as different directional images are individually binarized and recombined towards the end.

### 4.3. Comparison with State-of-the-Art

**Figure 9:**
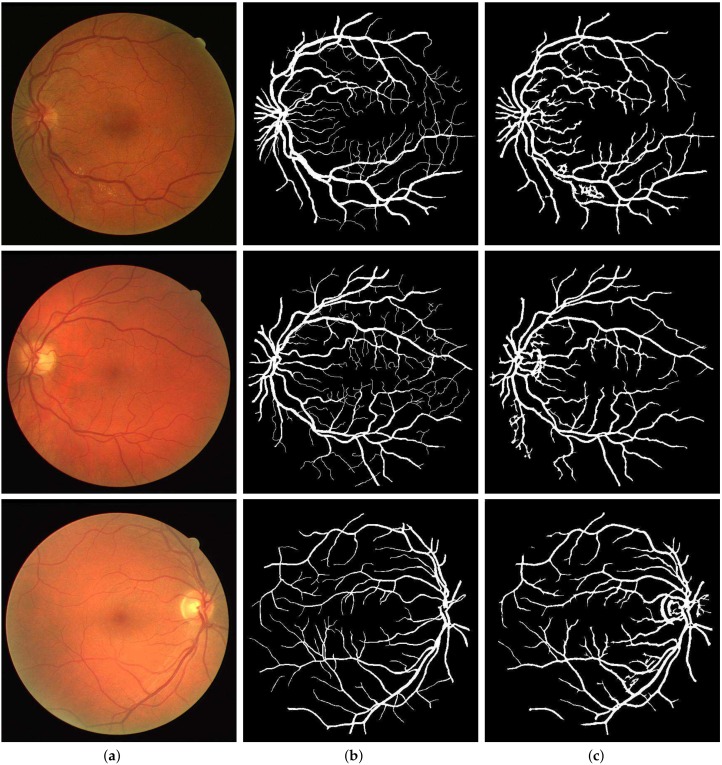
Segmentation results of DRIVE: (**a**) Color fundus images 3, 5 and 18; (**b**) Ground truths; (**c**) Binarization results.

**Figure 10:**
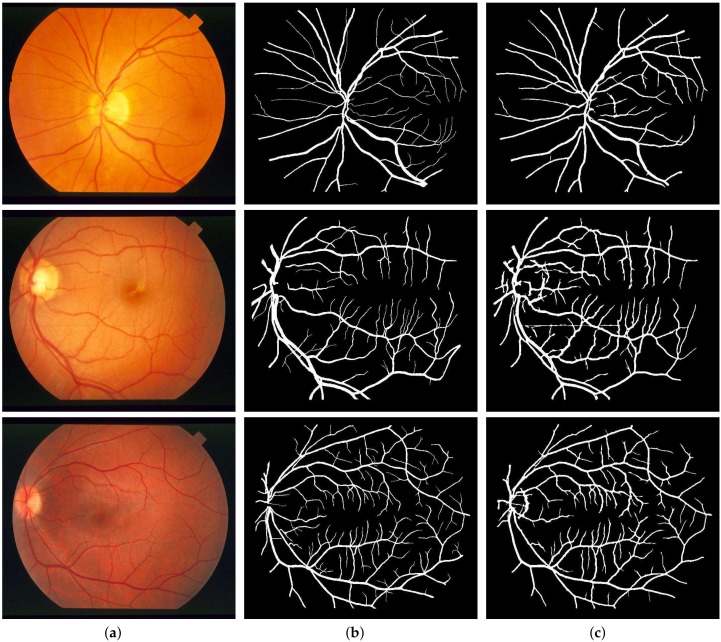
Segmentation results of STARE: (**a**) Color fundus images 12, 14 and 17; (**b**) Ground truths; (**c**) Binarization results.

**Figure 11:**
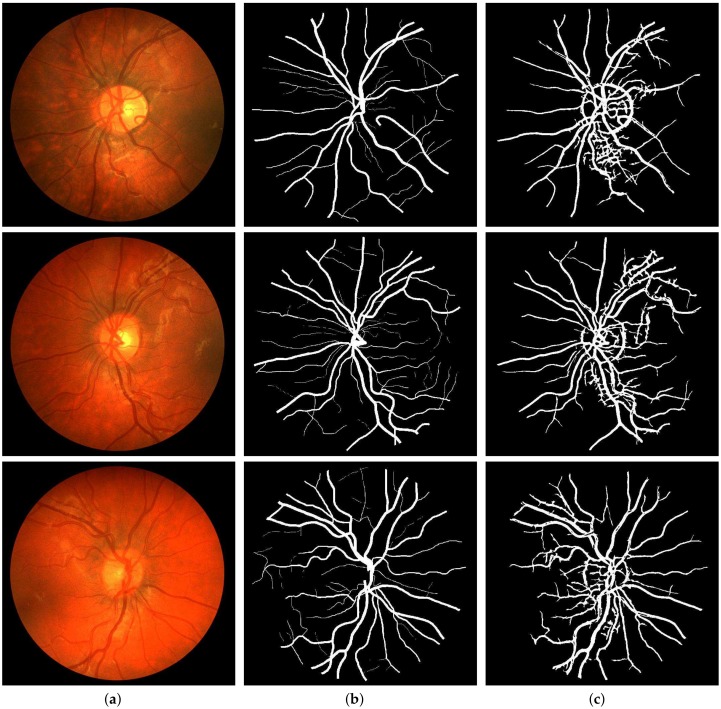
Segmentation results of CHASE_DB1: (**a**) Color fundus images 1, 5, and 10; (**b**) Ground truths; (**c**) Binarization results.

For the quantitative performance evaluation of our method, testing was carried out on the DRIVE, STARE, and CHASE_DB1 datasets with segmentation results as shown in Figure 9, Figure 10 and Figure 11, respectively. Performance measures such as Sn, Sp, and Acc for these databases are listed separately and compared with those of state-of-the-art techniques in Table 1, Table 2 and Table 3. Also, the computation time for the proposed method on MATLAB 2018b, comes out to 5 seconds per image on a Core i7 CPU (2.21 GHz, 16 GB RAM).

**Figure 12:**
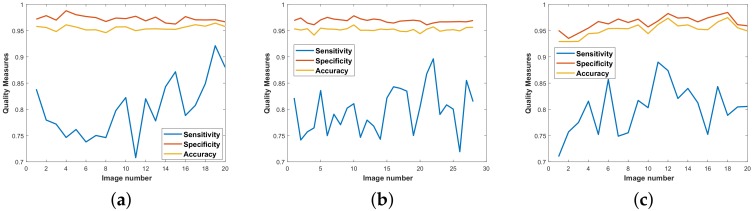
Quality measures for datasets: (**a**) DRIVE; (**b**) CHASE; (**c**) STARE.

It can be observed from these results that the proposed strategy of using directional multi-scale line detectors yields performance parameters that are at par with all the top-performing techniques in their respective categories. Most importantly, this method is equally potent irrespective of the benchmark dataset it is tested on, and produces all-round efficient results as shown in the quality measure graphs in Figure 12.

For the DRIVE database, we obtained 0.8043, 0.9730, and 0.9553 for Sn, Sp, and Acc, respectively. The Sn is the highest in its category and shows considerable enhancement over the second-best Aguiree [37], which exhibited a Sn of 0.7854. The highest Sn score is 0.92 for image 19 and lowest score is 0.71 for image 11. The highest Sp score is 0.99 for image 4 while the best Acc is 0.97 for image 19. Talking about Sp, our method just takes the edge over Zhang [34] with 0.9725, but loses the first place to Karn [36] with a Sp of 0.98. The same hybrid active contour technique by Karn [36] scores the highest Acc of all methods (0.97) with our method in second place. It is pertinent to note here that Karn [36] achieved these numbers at the cost of the method’s Sn and also rounded-off his performance parameters in his report to a decimal place accuracy of 2.

For the STARE database having the more challenging pathological images, we obtained averaged scores of 0.8011,0.9694, and 0.9545 for Sn, Sp, and Acc, respectively. The Sn of our method is in second place behind the method by Neto [35], having an impressive score of 0.8344 but his algorithm scored such a high Sn mark by sacrificing the Sp and Acc measures for his method. The highest Sn score for STARE is 0.89 for image 11, and lowest score is 0.71 for image 1. The best Sp score is 0.98 for image 12 while the highest Acc is 0.97 for image 18. The average Sp is almost at par with the first position orientation scores method by Zhang [34] at 0.9758. The average Acc measure of our method lies in third place close behind Karn [36] and Zhang [34] with Acc scores of 0.96 and 0.9554, respectively. Overall, our method consistently ranks in the top tier of all unsupervised vessel segmentation techniques for the STARE dataset.

Considering the CHASE_DB1 dataset, our proposed algorithm reports the highest Sn score which is a significant improvement upon the second place Karn [36] at 0.78. The highest Sn score for this dataset is 0.90 for image 21 and lowest score is 0.71 for image 26. The highest Sp score is 0.98 for image 10 while the best Acc is 0.96 for images 1 and 22. The average Sp score of our method is 0.9697, which barely loses out to the top performing technique in the category by Karn [36] at 0.97. The average Acc of our method stands at second position with a value of 0.9528, tagging behind top-performing algorithm by Karn [36] at 0.97. Comparatively, our directional multi-scale line detector exhibits all-round performance on a variety of databases and performs similar or sometimes superior when compared with the supervised learning algorithms.

## 5. Conclusions

In this paper, the use of directional multi-scale line detectors was proposed for segmenting directional vessel images extracted from DFB. The evaluation of this technique on three publicly available datasets suggested that this technique not only yielded balanced and robust performance parameters under difficult testing environments, but also competed with supervised learning techniques which are much more computationally intensive. This computational flexibility also gave this technique great leverage while working on considerably large databases with retinal disorders and abnormalities. The intricate geometry of retinal vessels meant that special methods be adopted to successfully segment troublesome areas such as parallel vessels and vessel crossings. The use of an array of directional images acted upon by a directional detector and binarization, helped this method achieve superior sensitivity results of 0.8043 and 0.7974 for the DRIVE and CHASE_DB1 datasets while maintaining decent specificity and accuracy scores.

The potential future research directions of this work include the exploration of combining the directional filters with contrast enhancement and noise cancellation techniques to optimize the performance and reduce the classification inaccuracies during the segmentation process.

## Figures and Tables

**Table 1:** Comparison with state-of-the-art techniques on the DRIVE database.

Type	Methods	Year	Sn	Sp	Acc	AUC
Supervised methods	Ricci [9]	2007	N.A	N.A	0.9595	0.9633
	Li [14]	2016	0.7569	0.9816	0.9527	0.9738
	Orlando FC [15]	2017	0.7893	0.9792	N.A	0.9507
	Orlando UP [15]	2017	0.7076	0.9870	N.A	0.9474
	Dasgupta [16]	2017	0.9691	0.9801	0.9533	0.9744
	Alom [17]	2018	0.7792	0.9813	0.9556	0.9784
	Oliveira [18]	2018	0.8039	0.9804	0.9576	0.9821
	Li [20]	2019	0.8349	N.A	0.9563	0.9157
	Jiang [21]	2019	0.7839	0.9890	0.9709	0.9864
	Guo [22]	2019	0.7891	0.9804	0.9561	0.9806
Unsupervised methods	Zhang [34]	2016	0.7743	0.9725	0.9476	0.9636
	Neto [35]	2017	0.7806	0.9629	0.8718	N.A
	Karn [36]	2018	0.78	0.98	0.97	0.88
	Aguiree [37]	2018	0.7854	0.9662	0.9503	N.A
	Sundaram [38]	2019	0.69	0.94	0.93	N.A
	Proposed	2019	0.8043	0.9730	0.9553	N.A

**Table 2:** Comparison with state-of-the-art techniques on the CHASE_DB1 database.

Type	Methods	Year	Sn	Sp	Acc	AUC
Supervised methods	Li [14]	2016	0.7507	0.9793	0.9581	0.9716
	Orlando FC [15]	2017	0.7277	0.9712	N.A	N.A
	Alom [17]	2018	0.7756	0.9820	0.9634	0.9815
	Oliveira [18]	2018	0.7779	0.9864	0.9653	0.9855
	Jiang [21]	2019	0.7839	0.9894	0.9721	0.9866
	Guo [22]	2019	0.7888	0.9801	0.9627	0.9840
Unsupervised methods	Zhang [34]	2016	0.7626	0.9661	0.9452	0.9606
	Karn [36]	2018	0.78	0.97	0.97	N.A
	Sundaram [38]	2019	0.71	0.96	0.95	N.A
	Proposed	2019	0.7974	0.9697	0.9528	N.A

**Table 3:** Comparison with state-of-the-art techniques on the STARE database.

Type	Methods	Year	Sn	Sp	Acc	AUC
Supervised methods	Ricci [9]	2007	N.A	N.A	0.9646	0.9680
	Li [14]	2016	0.7726	0.9844	0.9628	0.9879
	Orlando FC [15]	2017	0.7680	0.9738	N.A	N.A
	Orlando UP [15]	2017	0.7692	0.9675	N.A	N.A
	Alom [17]	2018	0.8298	0.9862	0.9712	0.9914
	Oliveira [18]	2018	0.8315	0.9858	0.9694	0.9905
	Li [20]	2019	0.8465	N.A	0.9673	0.9206
	Jiang [21]	2019	0.8249	0.9904	0.9781	0.9927
	Guo [22]	2019	0.8212	0.9843	0.9674	0.9859
Unsupervised methods	Zhang [34]	2016	0.7791	0.9758	0.9554	0.9748
	Neto [35]	2017	0.8344	0.9443	0.8894	N.A
	Karn [36]	2018	0.80	0.96	0.96	0.88
	Aguiree [37]	2018	0.7116	0.9454	0.9231	N.A
	Proposed	2019	0.8011	0.9694	0.9545	N.A
